# Radiological and Clinical Outcomes of Pediatric Patients With a Supracondylar Humerus Fracture Surgically Treated With Closed Reduction and Percutaneous Pinning

**DOI:** 10.7759/cureus.49358

**Published:** 2023-11-24

**Authors:** Oğuzhan Muslu, Tolgahan Cengiz, Şafak Aydın Şimşek, Alparslan Yurtbay, Davut Keskin

**Affiliations:** 1 Orthopaedics and Traumatology, Hatay Training and Research Hospital, Hatay, TUR; 2 Orthopaedics and Traumatology, Inebolu State Hospital, Kastamonu, TUR; 3 Orthopaedics and Traumatology, Faculty of Medicine, Ondokuz Mayıs University, Samsun, TUR; 4 Orthopaedics and Traumatology, Samsun Education and Research Hospital, Samsun, TUR

**Keywords:** pediatric fractures, flynn, gartland, percutaneous pinning, supracondylar humeral fracture

## Abstract

Objectives: It was aimed to evaluate the clinical and radiological results of patients operated on with closed reduction and pinning due to pediatric supracondylar humerus fractures.

Materials and methods: The radiological and clinical results of 100 patients operated on with closed reduction and percutaneous pinning for pediatric supracondylar humerus fractures in the Department of Orthopedics and Traumatology were examined between January 2015 and February 2022. Clinical results were evaluated by performing cosmetic and functional scores defined by Flynn. Closed reduction and percutaneous pinning techniques were used in surgical treatment.

Results: In our study, 59 patients were male (59%), and 41 were female (41%). The average age of all patients is 6.21 ± 2.85 years. According to the Gartland classification, 21 patients' fractures were type IIA (21%), 12 patients' fractures were type IIB (12%), 51 patients' fractures were type III (51%), and 16 patients' fractures were type IV (16%). The average number of pins used in the treatment is 2.55 ± 0.50. The elbow bearing angle of the operated side of the patients was an average of 6.53 ± 3.29 degrees, the humerocapitellar angle was an average of 41.97 ± 3.08 degrees, and the lateral humerocapitellar angle was an average of 50.17 ± 3.58 degrees. Fifty-one patients had stage 0 (51%), 23 patients had stage 1 (23%), 23 patients had stage 2 (23%), and three patients had stage 3 (3%) residual sagittal plane deformity. According to the Flynn criteria, 92 patients had excellent functional results (92%), seven patients had good results (7%), and one patient had fair results (1%). Regarding cosmetic results, 91 patients had excellent results (91%), six patients had good results (6%), and three patients had fair results (3%).

Conclusion: Supracondylar humerus fractures are common in children and can cause serious complications. Closed reduction and percutaneous pinning techniques are effective treatment methods in the treatment of displaced supracondylar humerus fractures.

## Introduction

Humeral supracondylar fractures account for 55-75% of elbow fractures in children and approximately 15% of all pediatric fractures [1,2]. The incidence of these fractures has been estimated at 177.3 per 100,000 [3]. The average age of application is six [2,4]. The non-dominant side is more affected [5]. This fracture has been reported to be more common in males [6]. However, some reports indicate a higher incidence in females [7]. Injury rates are higher on weekends and in the summer [4]. Supracondylar humerus fractures are characterized as extension or flexion type, depending on the distal fragment's displacement direction. Approximately 97-99% of supracondylar humerus fractures are extension-type [2]. Although several classifications have been proposed for supracondylar humerus fractures, the Gartland classification is the most widely used.

The incidence of vascular pathology in displaced supracondylar humerus fractures is reported to be 2-20% [8,9]. The hand is the best indicator of vascular status. Three situations are defined in vascular evaluation: normal hand, pink-pulseless hand, and white-pulseless hand. The treatment algorithm is determined according to these three conditions [9]. The most common neurological pathology is anterior interosseous nerve (AIN) damage, followed by radial nerve symptoms. The least common neurological injury is ulnar nerve damage. In a meta-analysis study including more than 5,000 fractures, the overall rate of traumatic neuropraxia was 11.3% [10].

Standard AP and accurate lateral radiographs of the elbow are usually sufficient to characterize the fracture. Since most classifications and treatment algorithms are based on degrees of extension and flexion displacement, the actual lateral view of the elbow is important. Nonoperative treatment of supracondylar humerus fractures is indicated in unstable Gartland type I fractures that do not involve neurovascular pathology [11]. Today, closed reduction and percutaneous pinning are the gold standard treatment for all displaced fractures [12]. The ever-increasing need for postoperative analgesia is the most critical indicator of compartment syndrome in children. If there is no pain, or compartment or neurovascular problems, the child can be discharged on the first day after surgery [11].

Treatment of these fractures is challenging due to the complex and difficult-to-understand anatomy of the elbow. The physician's experience and knowledge are associated with positive treatment results and reduced complication rates. They should be considered because they are frequently seen and cause complications such as accompanying neurovascular injuries, the possibility of developing compartment syndrome, decreased joint range of motion, and sagittal-coronal plane deformities [13]. Modern techniques for treating supracondylar humeral fractures in children have significantly reduced the rates of malunion and compartment syndrome [8].

The purpose of this study is to retrospectively evaluate the functional and radiological results of pediatric patients treated with closed reduction and percutaneous pinning due to supracondylar humerus fractures and compare them with the literature.

## Materials and methods

In this study, 100 patients who were diagnosed with pediatric supracondylar humerus fracture by examination and an imaging method, who were operated on by a closed reduction and percutaneous pinning method, who came for the last follow-up examination and met the inclusion criteria between January 2015 and February 2022, were examined in a tertiary university hospital. Clinical and radiological results were evaluated retrospectively. Ethics committee approval for the study was received from Ondokuz Mayıs University Faculty of Medicine Ethics Committee with OMUKAEK number 2022/170 and application number 2022000170-2.

Patients with extension Gartland type II, type III, and type IV pediatric supracondylar humerus fractures, who were operated on by closed reduction and percutaneous pinning, whose preoperative and postoperative radiographs were available in the hospital database and who came for the last follow-up examination, were included in the study. Gartland type I pediatric supracondylar humerus fractures, flexion type fractures, open fractures, patients operated on with open reduction, pathological fractures, patients with metabolic and congenital disorders, patients over 18 years of age, patients with lack of records and imaging during follow-up, and patients who did not come for a control examination were not included in the study.

Patient records who applied to the Orthopedics and Traumatology Department were accessed by scanning the hospital information system archive records. Protocol numbers of the patients, name-surname, gender, age, age at the time of surgery, dominant extremity, fractured extremity, number of hours following the injury, fracture type, presence of complications, relevant neurovascular status, functional and cosmetic scoring values according to the criteria defined by Flynn measurements of both elbow joint ranges of motion, bilateral carrying angles, Baumann angles, and humerocapitellar and lateral humerocapitellar angles were evaluated and recorded on the prepared forms.

At the last follow-up examination, the patient's elbow bearing angles and joint range of motion were measured using a goniometer using the McRae method. While measuring the elbow carrying angle, the goniometer was placed with the elbow in extension and the forearm in supination, with the center in the antecubital region. A goniometer was placed with the lateral epicondyle as its center point to measure the joint range of motion. Afterward, the degrees of full extension and full flexion were recorded while the forearm was in supination. The difference in the values measured in both elbows was found. These differences were evaluated according to functional and cosmetic criteria defined by Flynn. In addition, the patients' comparative elbow AP and lateral radiographs were taken at the last follow-up examination. Both elbow humerocapitellar angles, rotational deformity, and residual sagittal plane deformity, were determined with the digital measurement method available in picture archiving and communication systems (PACS).

The Statistical Product and Service Solutions (SPSS, version 26) (IBM SPSS Statistics for Windows, Armonk, NY) program was used for statistical analysis. Descriptive analyses were performed for the general characteristics of the groups. Categorical variable data were expressed as n (%), and continuous variable data were expressed as mean ± standard deviation. One-way analysis of variance and the significance of the difference between two means tests were used when comparing the means of quantitative variables between groups. When the comparison of quantitative data did not comply with normal distribution, the Mann-Whitney U test was performed. In comparing quantitative data, Student's t-test was used when comparing normally distributed findings between two groups. Chi-square test and Crosstabs were used to evaluate the relationship between qualitative data. P values less than 0.05 were considered statistically significant.

## Results

In our study, 59 patients were male (59%), and 41 were female (41%). The average age of all patients is 6.21 ± 2.85 years. The average age of male patients is 6.78 ± 3.14 years, and that of female patients is 5.40 ± 2.13 years. It was observed that fractures developed in the left elbow of 61 patients (61%) and in the right elbow of 39 patients (39%). The dominant extremity of 32 patients (32%) and the non-dominant extremity of 68 patients (68%) were fractured. According to the Gartland classification, 21 patients' fractures were type IIA (21%), 12 patients' fractures were type IIB (12%), 51 patients' fractures were type III (51%), and 16 patients' fractures were type IV (16%). The average number of pins used in the treatment is 2.55 ± 0.50. The elbow bearing angle of the operated side of the patients was an average of 6.53 ± 3.29 degrees, the humerocapitellar angle was an average of 41.97 ± 3.08 degrees, and the lateral humerocapitellar angle was an average of 50.17 ± 3.58 degrees.

**Figure 1:**
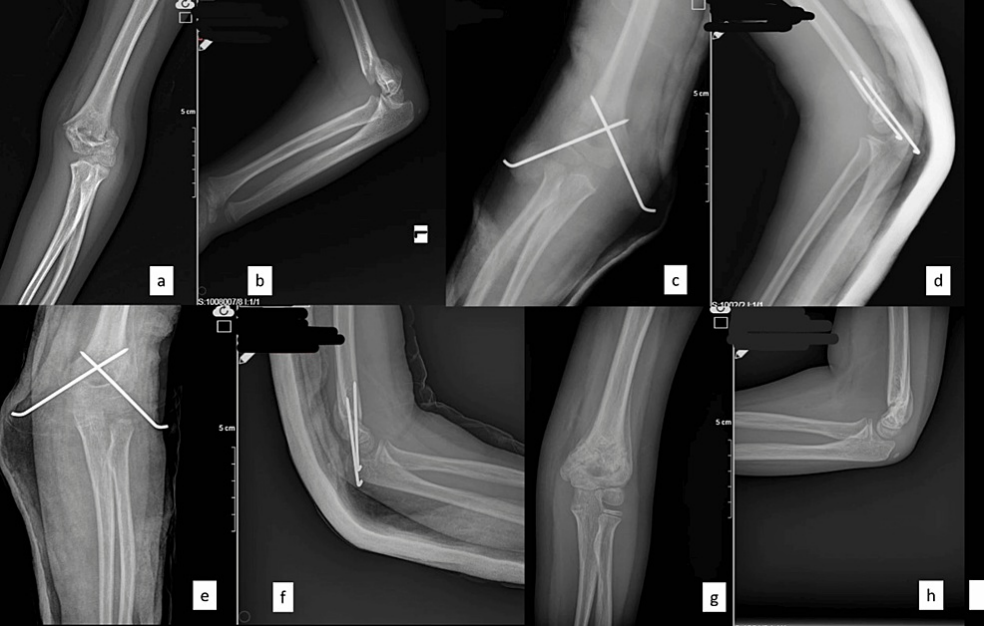
a) Preoperative elbow AP X-ray. b) Preoperative elbow lateral X-ray. c) Postoperative first-day elbow AP X-ray. d) Postoperative first-day elbow lateral X-ray. e) Postoperative third-week elbow AP X-ray. f) Postoperative third-week elbow lateral X-ray. g) Postoperative sixth-week elbow AP X-ray. h) Postoperative sixth-week elbow lateral X-ray

**Figure 2:**
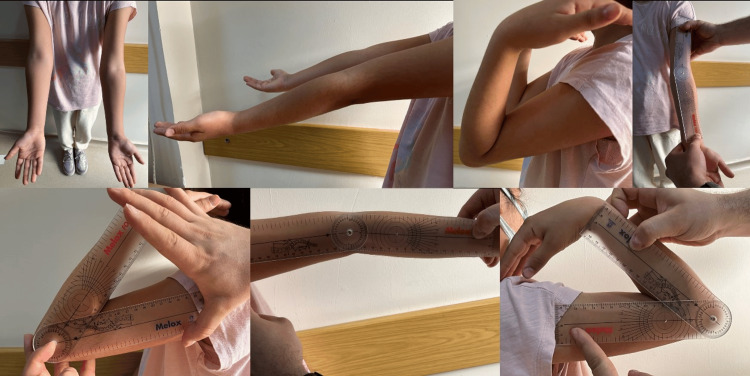
In the last follow-up examination, using a goniometer with the McRae method, the carrying angle, extension angle, and flexion angle of both elbows were measured

**Figure 3:**
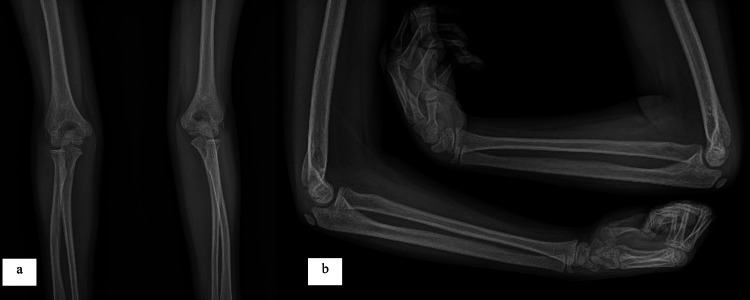
a) Last follow-up examination (second year) comparative elbow AP X-ray. b) Last follow-up examination (second year) comparative elbow lateral X-ray

An example of a patient who was operated on in our clinic due to a supracondylar humerus fracture is given in the following figures. An eight-year and three-month-old female had presented to the emergency department after a fall while roller skating. She had a supracondylar humerus fracture, Gartland type IIB, on her left elbow. Her neurovascular examination was intact. She underwent surgery using a closed reduction and percutaneous pinning technique. Two pins were used with a cross-pin configuration (Figure 1). Three weeks later, the cast was removed, and exercises were started after three weeks. In the sixth week, the wires were removed. The patient has been under follow-up for two years. In the last check-up examination (Figure 2), the range of motion in the operated side joint was 148 degrees. The elbow valgus angle was 11 degrees. The Baumann angle was 66.2 degrees. There were no sagittal plane deformities (0-33%) (Figure 3). The Flynn functional and cosmetic results were excellent.

**Figure 4:**
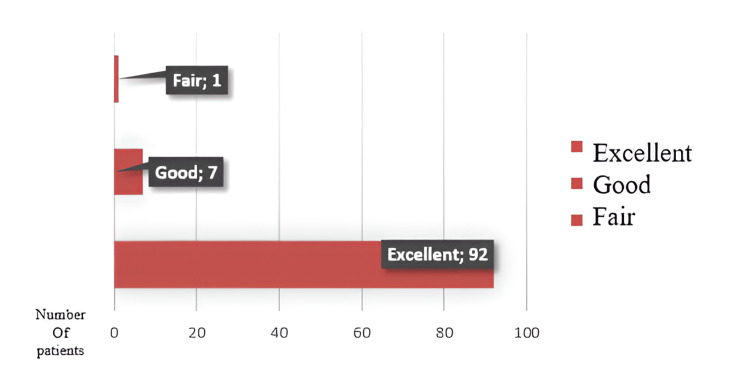
Flynn’s criteria functional results

**Figure 5:**
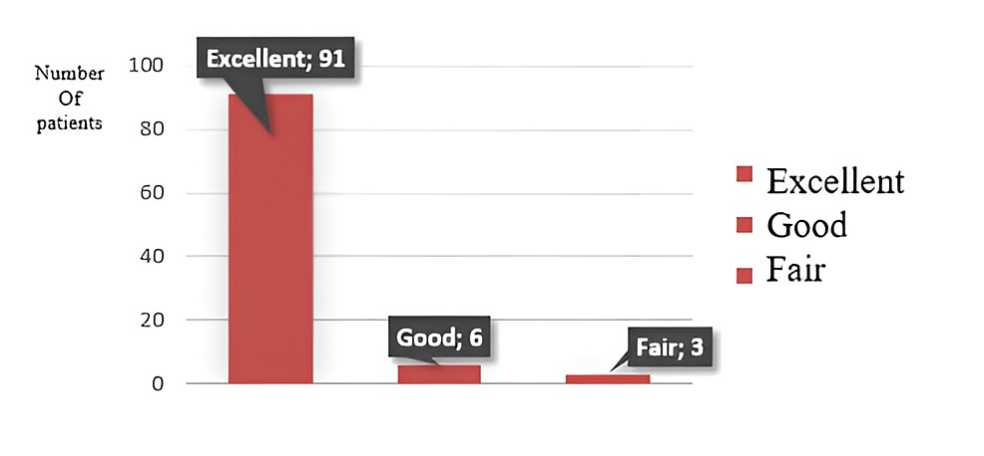
Flynn’s criteria for cosmetic results

The average humerocapitellar angle is 41.97 ± 3.08 degrees on the operated side and 41.45 ± 3.16 degrees on the average side. Residual sagittal plane deformity on the operated side was between 0% and 33% (Stage 0) in 51 patients, between 33% and 66% (Stage 1) in 23 patients, between 66% and 100% (Stage 2) in 23 patients, and above 100% in three patients (Stage 3). According to the Flynn criteria, in terms of functional results, 92 patients had excellent results, seven patients had good results, and one patient had fair results. Regarding cosmetic results, 91 patients had excellent results, six had good results, and three had acceptable results (Figures 4-5).

Specifically, 95.2% of patients with Gartland type IIA fractures and 58.3% with Gartland type IIB fractures have Grade 0 residual sagittal plane deformity. Stage 1 residual sagittal plane deformity developed in 33.3% of patients with Gartland type IIB fractures and 33.3% with Gartland type III fractures. Moreover, 19.6% of patients with Gartland type III fractures and 75% with Gartland type IV fractures have Grade 2 residual sagittal plane deformity. All three patients with Stage 3 sagittal plane deformity had Gartland type IV fractures. No sagittal plane deformity is more significant than 66% observed in any of the patients with Gartland type IIA fractures. No sagittal plane deformity greater than 100% was observed in patients with Gartland type IIA, IIB, and III fractures. All patients with Gartland type IIA and type IIB fractures had excellent results on the Flynn functional criteria (100%). The outcome was excellent in 94.1% of patients with Gartland type III fractures and good in 5.9%. Of the patients with Gartland type IV fractures, the outcome was excellent in 68.8%, good in 25%, and fair in 6.3%. All patients with Gartland type IIA fractures had excellent results according to the Flynn cosmetic criteria (100%). Two patients with moderate results according to the Flynn cosmetic criteria were Gartland type IV, and one patient had Gartland type III fracture.

The average joint range of motion on the operated side in patients with rotational deformity is 141.50 ± 6.40 degrees, and in patients without rotational deformity, it is 151.86 ± 5.80 degrees. The average difference in the range of motion of both elbow joints in patients with rotational deformity is -6.17 ± 3.49 degrees, and in patients without rotational deformity is -1.14 ± 1.85 degrees. According to the Flynn cosmetic criteria, 96.7% of patients with excellent results do not have a rotational deformity, and 100% of patients with good or fair cosmetic results have rotational deformity (Table 1).

**Table 1:** Evaluation of quantitative data based on the presence of rotational deformity HK: Humerokapitellar, LHK: Lateral humerokapitellar

Variable	Deformity (+)	Deformity (-)	p value
Operated side range of motion	141.50±6.40^o^	151.86±5.80^o^	0.000
Range of motion difference	-6.17±3.49^o^	-1.14±1.85^o^	0.000
Valgus angle difference	-5.33±5.42^o^	-0.32±1.13^o^	0.000
Operated side valgus angle	3.67±6.24^o^	6.92±2.47^o^	0.008
Baumann angle	69.22±2.70^o^	68.79±1.95^o^	0.480
Operated side HK angle	40.16±4.06^o^	41.63±3.01^o^	0.367
Operated side LHK angle	51.75±5.10^o^	49.95±3.31^o^	0.119
Surgery duration	73.50±47.57 min	62.85±28.03 min	0.592
Age	7.31±3.16 years	6.06±2.79 years	0.216
Time of mobilization	4.00±0.85 week	3.91±0.77 week	0.800
Time of pin removal	6.17±0.94 week	5.85±0.56 week	0.356

While 95.7% of the patients with perfect results on the Flynn functional criteria do not have rotational deformity, 100% of the patients with excellent or fair functional results have a rotational deformity. Rotational deformity occurred in all three patients with sagittal plane deformity over 100%. While rotational deformity developed in 30.4% of 23 patients with Stage 2 sagittal plane deformity, none of the 23 patients with Stage 1 had rotational deformity. In 3.9% of 51 patients with sagittal plane deformity Stage 0, rotational deformity occurred. The average joint range of motion on the operated side was 152.73 ± 5.20 in patients with residual sagittal plane deformity Stage 0, while it was 151.91 ± 5.71 in Stage 1, and it is 146.52 ± 7.29 degrees in Stage 2 and 136.33 ± 1.53 degrees in Stage 3. The difference in range of motion of both elbow joints averaged -1.22 ± 1.45 in patients with residual sagittal plane deformity in Stage 0, -1.44 ± 1.67 in Stage 1; It is -2.21 ± 3.78 degrees in Stage 2, and -9.33 ± 3.51 degrees in Stage 3 (Table 2).

**Table 2:** Evaluation of the quantitative data based on sagittal plane deformity HK: Humerokapitellar, LHK: Lateral humerokapitellar

Variable	Stage 0	Stage 1	Stage 2	Stage 3	p value
Operated side range of motion	152.73±5.20^o^	151.91±5.71^o^	146.52±7.29^o^	136.3±1.53^o^	0.000
Range of motion difference	-1.22±1.45^o^	-1.44±1.67^o^	-2.22±3.78^o^	-9.33±3.51^o^	0.006
Valgus angle difference	-0.63±1.82^o^	0.00±1.31^o^	-1.22±2.61^o^	-4.00±5.00^o^	0.002
Operated side valgus angle	6.78±2.82^o^	6.70±2.14^o^	7.17±2.82^o^	-10.67±4.16^o^	0.028
Baumann angle	68.74±1.65^o^	69.57±1.55^o^	68.09±2.64^o^	70.63±4.23^o^	0.162
Operated side HK angle	42.55±2.84^o^	41.50±3.01^o^	41.44±3.38^o^	39.67±4.62^o^	0.153
Operated side LHK angle	49.65±3.12^o^	49.29±3.31^o^	52.17±4.08^o^	50.30±5.15^o^	0.051
Age	6.32±3.08 years	5.67±2.62 years	6.05±2.30 years	9.90±2.46 years	0.109
Time of mobilization	3.90±0.76 week	3.78±0.67 week	4.13±0.92 week	3.67±0.58 week	0.636
Time of pin removal	5.86±0.60 week	5.78±0.52 week	6.09±0.73 week	5.67±0.58 week	0.289
Surgery duration	62.65±25.90 min	62.09±27.15 min	63.48±32.44 min	110.00±86.60 min	0.671

The sagittal plane deformity is over 100% in 66.7% of the patients whose results are average according to the Flynn cosmetic criteria. The sagittal plane deformity is at most 100% in any of the patients with perfect results according to the Flynn cosmetic criteria. The sagittal plane deformity is above 100% in 100% of the patients with fair results on the Flynn functional criteria and in 28.6% of the patients with good results. The sagittal plane deformity is at most 100% in any of the patients with perfect results on the Flynn functional criteria.

## Discussion

The residual sagittal plane deformity is defined as the distance between the centre of the capitellum and the anterior humeral line (AHL) divided by the radius of the capitellum. This value was determined as 0% when the AHL crossed the center of the capitellum and 100% when the AHL crossed the anterior or posterior surface of the capitellum. Sagittal deformity between 0% and 33% is defined as Stage 0. Deformity between 33% and 66% is defined as Stage 1 (mild), between 66% and 100% is defined as Stage 2 (moderate), and above 100% is defined as Stage 3 (severe) deformity. In the study conducted by Silva et al. [14], the residual sagittal deformity was observed in approximately 10% of the patients. Additionally, 95.2% of our patients with Gartland type IIA fractures have Stage 0, and 75% of our patients with Gartland type IV fractures have Stage 2 residual sagittal plane deformity. All three cases with Stage 3 deformity were patients with Gartland type IV fractures. No sagittal plane deformity was observed in more than 66% of patients with Gartland type IIA fractures. Our result was lower than the rate reported by Silva et al. [14]. This shows that our clinical outcomes are good. According to the data obtained from our study, the incidence of more severe residual sagittal plane deformity increases in advanced fracture types (p<0.001). This situation is likely caused by the direct proportion between the increasing severity of the fracture and the difficulty of achieving anatomical reduction. Additionally, the increased severity of the fracture may have caused this condition by further impairing the blood supply of the distal humerus.

Flynn defined a number of criteria to evaluate cosmetic and functional results. These criteria were determined as excellent, good, moderate, and poor according to the angular difference between both extremities. Elbow bearing angle is used for cosmetic criteria, and joint range of motion is used for functional criteria [15]. Of the 100 patients evaluated in our study, Flynn's functional results were excellent in 92%, and cosmetic criteria results were excellent in 91%. The Flynn functional criteria results of all patients with Gartland type IIA and type IIB fractures were excellent (100%). The outcome is remarkable in 94.1% of patients with Gartland type III fractures and 68.8% with Gartland type IV fractures. The excellent functional outcome rate decreases as the fracture's severity increases (p=0.012).

All patients with Gartland type IIA fractures had excellent results according to the Flynn cosmetic criteria (100%). The outcome is remarkable in 96.1% of patients with Gartland type III fractures and 68.8% with Gartland type IV fractures. As the fracture stage progresses, the rate of excellent cosmetic results decreases (p=0.011). The rates and distributions we found were mainly similar to the results in the study of 116 cases conducted by Mazda et al. [16]. Our results were highly satisfactory according to the Flynn criteria because we applied the surgical technique correctly and followed up with the patients well. The humerocapitellar angle of our patients on the operated side, average Gartland type IIA: 43.06 ± 2.19; type IIB: 40.34 ± 2.22; type III: 42.32 ± 2.81; and type IV: 40.63 ± 3.52 degrees. The average results for all fracture types were within the normal range [17]. A statistically significant difference was found between type IIA and type IV (p=0.032). No publication has been found in the literature comparing the humerocapitellar angles of Gartland fracture types. In our study, the average humerocapitellar angle of all patients on the operated side was 41.97 ± 3.08 degrees. It is similar to the result in the study of 1,518 cases conducted by Boden et al. [18].

Stage 0 residual sagittal plane deformity was observed in 51 of 100 patients, Stage 1 in 23 patients, Stage 2 in 23 patients, and Stage 3 in three patients. Our distribution was found to be similar to the distribution in the study by Silverstein et al. [19]. There is limited literature regarding the long-term functional consequences of sagittal plane deformity in pediatric supracondylar fractures [17,20]. In their study, Silverstein et al. did not find a significant relationship between the degree of sagittal deformity and joint range of motion and humerocapitellar angle [19]. In a study by Persiani et al. [21], no significant relationship was found between sagittal alignment, joint range of motion, and humerocapitellar angle. Additionally, their study found an average of 10 degrees of difference in the flexion-extension arc of motion on the operated side compared to the opposite extremity [21]. In their study, Simanovsky et al. [20] could not specify acceptable residual sagittal deformity limits due to the small number of patients. They stated that patients with insufficient remodeling in the sagittal plane had flexion limitations [20]. In our study, the average joint range of motion on the operated side was 152.73 ± 5.20 in patients with Stage 0, respectively, along with Stage 1: 151.91 ± 5.71; Stage 2: 146.52 ± 7.29 degrees; and Stage 3: 136.33 ± 1.53 degrees (p<0.001). All groups have a movement arc that will not functionally affect their daily activities. The average difference in range of motion of both elbow joints in patients with sagittal plane deformity Stage 0 was -1.22 ± 1.45; -1.44 ± 1.67 in Stage 1, and it was -2.21 ± 3.78 degrees in Stage 2 and 9.33 ± 3.51 degrees in Stage 3 (p=0.006). We attribute this to the progression of the deformity level as the severity of the fracture type increases, similar to the literature [22]. These results show a significant relationship between sagittal plane deformity and range of motion. None of the patients whose results were perfect according to the Flynn cosmetic and functional criteria had sagittal plane deformity above 100%. These results were found to be statistically significant (p<0.001). There is a greater risk of sagittal plane deformity in severe-type fractures. Generally, cosmetic and functional results worsen in advanced-stage fractures [16,23]. In a study by Tuomilehto et al., the Flynn criteria results were worse in advanced-stage fractures [24].

There are few studies in the literature evaluating the presence of rotational deformity. In a study conducted by Henderson et al., they made radiographic measurements and formulated this to define the presence of rotational deformity [25]. Using the Henderson technique, our study divided the patients into two groups: patients with rotational deformity below 10 degrees without deformity and patients with deformity above 10 degrees. The functional and cosmetic results of all 88 patients without deformity were excellent (p<0.001). As the fracture severity increases, reduction and stabilization difficulties occur. Difficulty in reduction and stabilization in advanced fracture types increases the risk of deformity development. As a result, we argue that deformity develops more in severe fractures, which negatively affects the Flynn criteria.

In all three patients with Stage 3 sagittal plane deformity, rotational deformity was observed in 30.4% of 23 patients with Stage 2 and 3.9% of 51 patients with Stage 0. On the other hand, no rotational deformity was observed in any of the 23 patients with Stage 1 (p<0.001). Skibo et al. stated that external rotation of the condyle will move the capitellum posteriorly in the sagittal plane, disrupting the sagittal alignment [26]. Patients with advanced sagittal deformity appear to be accompanied by more rotational deformity. We attribute this to the increasing difficulty of reduction with increasing fracture severity and further deterioration of the nutrition of the distal humerus. The average joint range of motion on the operated side of patients with rotational deformity was 141.50 ± 6.40 degrees, and that of patients without rotational deformity was 151.86 ± 5.80 degrees (p<0.001). The average difference in the range of motion of both elbow joints in patients with rotational deformity is 6.17 ± 3.49 degrees, and in patients without rotational deformity, it is 1.14 ± 1.85 degrees (p<0.001). Both groups have a movement arc that will not functionally affect their daily activities [22]. Silverstein et al. did not find a significant relationship between AHL, rotational deformity, and joint range of motion in their study [19]. Our results are similar to those between fracture types and range of motion. We attribute this to the deformity level's progression as the fracture type's severity increases.

The main limiting factor of our study was the inclusion of only patients treated with closed reduction and percutaneous pinning. In this way, open reduction could not be compared with closed reduction and open fractures with closed fractures. Furthermore, there was no opportunity to evaluate the analyzed data in larger and different case series. In addition, not comparing the cross-pin configuration and lateral pin configuration caused a deficiency in terms of evaluation. Another shortcoming of our study is that there was only one observer for radiologic evaluation, digital measurements, clinical evaluation, and reproducible manual goniometer measurements. This resulted in an increased risk of bias.

## Conclusions

Supracondylar fractures of the humerus are a very common disease in children, but publications evaluating the sagittal plane deformities and rotational deformities seen in these fractures are very limited. More studies on this subject are needed with large patient groups with long-term follow-up. Our study will contribute to the literature on this subject. Finally, the closed reduction and percutaneous pinning method used in treating closed and displaced extension-type fractures in children is a practice that gives satisfactory results when performed appropriately.
